# Beat-to-Beat Variation in Periodicity of Local Calcium Releases Contributes to Intrinsic Variations of Spontaneous Cycle Length in Isolated Single Sinoatrial Node Cells

**DOI:** 10.1371/journal.pone.0067247

**Published:** 2013-06-27

**Authors:** Oliver Monfredi, Larissa A. Maltseva, Harold A. Spurgeon, Mark R. Boyett, Edward G. Lakatta, Victor A. Maltsev

**Affiliations:** 1 Laboratory of Cardiovascular Science, National Institute on Aging - Intramural Research Program, National Institutes of Health, Baltimore, Maryland, United States of America; 2 Institute of Cardiovascular Sciences, University of Manchester, Manchester, United Kingdom; Brigham & Women's Hospital - Harvard Medical School, United States of America

## Abstract

Spontaneous, submembrane local Ca^2+^ releases (LCRs) generated by the sarcoplasmic reticulum in sinoatrial nodal cells, the cells of the primary cardiac pacemaker, activate inward Na^+^/Ca^2+^-exchange current to accelerate the diastolic depolarization rate, and therefore to impact on cycle length. Since LCRs are generated by Ca^2+^ release channel (i.e. ryanodine receptor) openings, they exhibit a degree of stochastic behavior, manifested as notable cycle-to-cycle variations in the time of their occurrence.

**Aim:**

The present study tested whether variation in LCR periodicity contributes to intrinsic (beat-to-beat) cycle length variability in single sinoatrial nodal cells.

**Methods:**

We imaged single rabbit sinoatrial nodal cells using a 2D-camera to capture LCRs over the entire cell, and, in selected cells, simultaneously measured action potentials by perforated patch clamp.

**Results:**

LCRs begin to occur on the descending part of the action potential-induced whole-cell Ca^2+^ transient, at about the time of the maximum diastolic potential. Shortly after the maximum diastolic potential (mean 54±7.7 ms, n = 14), the ensemble of waxing LCR activity converts the decay of the global Ca^2+^ transient into a rise, resulting in a late, whole-cell diastolic Ca^2+^ elevation, accompanied by a notable acceleration in diastolic depolarization rate. On average, cells (n = 9) generate 13.2±3.7 LCRs per cycle (mean±SEM), varying in size (7.1±4.2 µm) and duration (44.2±27.1 ms), with both size and duration being greater for later-occurring LCRs. While the timing of each LCR occurrence also varies, the LCR period (i.e. the time from the preceding Ca^2+^ transient peak to an LCR’s subsequent occurrence) averaged for all LCRs in a given cycle closely predicts the time of occurrence of the next action potential, i.e. the cycle length.

**Conclusion:**

Intrinsic cycle length variability in single sinoatrial nodal cells is linked to beat-to-beat variations in the average period of individual LCRs each cycle.

## Introduction

The heart rate of healthy subjects exhibits complex variability that is not well understood. A loss of this complexity is associated with diverse pathological conditions [1]. While heart rate variability is partly effected by autonomic nervous system modulation, cardiac pacemaker cells also exhibit their own intrinsic variability in spontaneous action potential (AP) cycle length (CL) [2]. The present dogma is that intrinsic CL variability of cardiac pacemaker cells (i.e. single, isolated cells) is caused by stochastic opening/closing of membrane ion channels [2], [3]. The paradigm of cardiac pacemaker cell function, however, has recently shifted from a mainly electrophysiological description to a coupled system of two oscillators (or “clocks”) involving a voltage-dependent membrane clock, and an intracellular Ca^2+^ clock, centered around the sarcoplasmic reticulum (SR) [4].

In the absence of APs, the SR in cardiac ventricular cells generates spontaneous, stochastic local Ca^2+^ releases (dubbed “Ca^2+^ sparks”) via ryanodine receptors (RyRs, the SR Ca^2+^ release channel) [5]. The SR of sinoatrial node cells (SANC, the primary cardiac pacemaker cells) also spontaneously generates local Ca^2+^ releases (LCRs) in various cell loci in the absence of APs, which are larger in size compared to sparks in ventricular myocytes and importantly, are roughly periodic in occurrence [6]. Under normal conditions, SANC generate spontaneous APs due to spontaneous diastolic depolarization (DD). An AP causes a global cytosolic Ca^2+^ transient and *en masse* Ca^2+^ depletion of the SR [7] that temporally interrupts generation of LCRs. Subsequent spontaneous diastolic RyR activation following replenishment of SR Ca^2+^ by its ATPase, ‘SERCA’, generates the appearance of partially synchronized LCRs. These LCRs activate an inward diastolic Na^+^/Ca^2+^ exchanger (NCX) current [8], [9] that, in turn, contributes to spontaneous DD, and initiates the generation of the next AP.

The time between the preceding AP-induced *en masse* RyR Ca^2+^ release (i.e. the Ca^2+^ transient (CaT) peak) and the subsequent appearance of a diastolic LCR in SANC is termed the ‘LCR period’. Since LCRs are generated by Ca^2+^ release channel (RyR) openings, individual LCRs exhibit a degree of stochasticity, manifested as notable cycle-to-cycle variations in their time of occurrence [6]. We tested the hypothesis that beat-to-beat variation in spontaneous AP CL in SANC is contributed by beat-to-beat variation in ensemble LCR period. To test this hypothesis, we used a two-dimensional (2D) camera to image LCRs (in selected cells, we simultaneously recorded APs via perforated patch), and for the first time, directly tested how the characteristics of the entire ensemble of LCRs impacts upon the DD and each CL on a beat-to-beat basis.

## Methods

### Single SANC Isolation

Single, spontaneously beating, spindle-shaped SANC were isolated from 8–12 week old male New Zealand White rabbit hearts as previously described [10].

### Ethics Statement

The study conformed to the Guide for the Care and Use of Laboratory Animals, published by the US National Institutes of Health. The experimental protocols were approved by the Animal Care and Use Committee of the National Institutes of Health (protocol # 034 LCS 2013). The rabbits weighed 1.8–2.5 kg and were deeply anaesthetized with sodium pentobarbital (50–90 mg/kg) injected to the central ear vein. The adequacy of anesthesia was monitored until reflexes to ear pinch and jaw tone were lost.

### 2D Ca^2+^ Dynamics Measurements in Single SANC

Ca^2+^ dynamics within isolated single SANC were measured by 2D imaging of fluorescence emitted by the Ca^2+^ indicator Fluo-4 using a high-speed Hamamatsu C9100-12 CCD camera (100 frames/second, with an 8.192 mm square sensor of 512×512 pixels resolution). This was mounted on a Zeiss Axiovert 100 inverted microscope (Carl Zeiss, Inc., Germany) with a x63 oil immersion lens and a fluorescence excitation light source (Sutter Instrument, LB-LS/Q17) housing a 175W xenon lamp. The light from the lamp was transferred to the fluorescence microscope via a liquid light guide (LLG, 2 meters, 3 mm diameter) to avoid vibrations from the light source. Fluo-4 fluorescence excitation (blue light, 470/40 nm) and emission light collection (green light, 525/50 nm) were performed using the Zeiss filter set 38 HE. Cells were loaded with 1.5 µM Fluo-4AM (Sigma-Aldrich) for 10 minutes at room temperature. Fluo-4AM was subsequently washed out of the chamber, and Ca^2+^ signals were measured within the ensuing 60 minutes at 35°C ±0.1°C. To avoid phototoxicity, Fluo-4 was excited only for short periods of time (<10s, see next section for details). Data acquisition was performed using SimplePCI (Hamamatsu Corporation, Japan). Only cells with a spontaneous cycle length of less than 500 ms (i.e. with spontaneous beating rate >2 Hz, close to that observed *in vivo*) were considered for further analysis. Movie S1 shows a representative high-speed camera recording of typical Ca^2+^ signal dynamics in SANC.

### Optimization of the Fluo-4 Loading and Excitation Procedure (Control Experiments)

**Figure 1:**
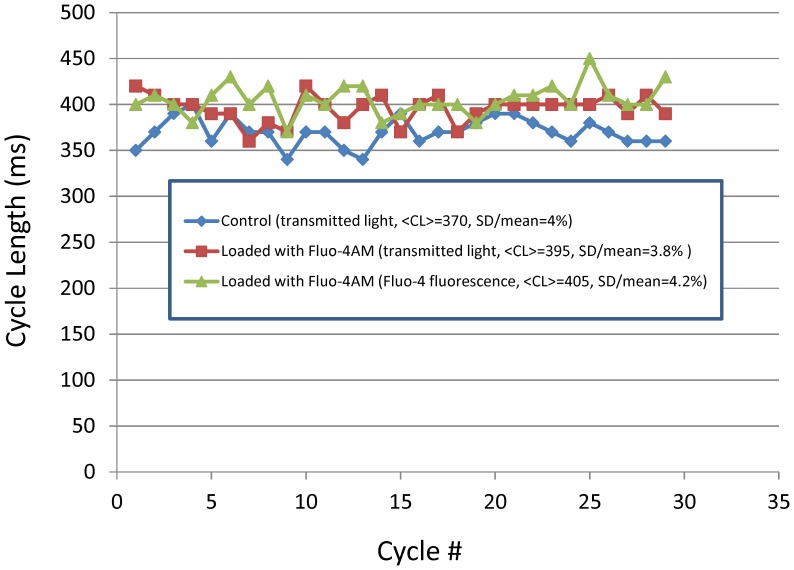
Fluo-4 loading and degradation does not alter intrinsic variation in spontaneous beating cycle length. Shown are the plots of 29 consecutive values of cycle length measured in a single SANC (cell #2 in Table 1) at 35°C under three separate conditions: before Fluo-4 loading (blue line), after Fluo-4 loading (red line), and during Fluo-4 excitation by blue light, as used for fluorescence measurements (green line).

Since SANC spontaneous beating rate and rhythm can be influenced by Fluo-4 loading (via its recognized effect on Ca^2+^ buffering) and also by Fluo-4 excitation (via Fluo-4 degradation resulting in photo-damage), we performed control experiments to ensure that our chosen experimental protocol did not substantially alter the intrinsic spontaneous CL and CL variability (the subject of the study). An example of such an experiment performed in one cell is illustrated in Figure 1. The complete results of the control experiments performed in 10 cells are provided in Table 1. Specifically, we measured CL and CL variability under three discrete conditions in each spontaneously beating SANC :

1. before cell loading with Fluo-4AM
2. after cell loading with Fluo-4AM in a low intensity transmitted red light (to prevent excitation and photo-damage by Fluo-4)
3. after cell loading with Fluo-4AM when fluo-4 was excited by blue light of 470/40 nm

**Table 1:** Intrinsic variation in spontaneous beating cycle length (CL) of rabbit SA node cells (assessed as SD/Mean, measured at 35°C) is not disturbed by our experimental conditions.

	Before Fluo-4AM loading			After Fluo-4AM loading			After Fluo-4AM loading		
	CL in red light			CL in red light			CL in blue light		
	Transmitted light			Transmitted light			Fluo-4 fluorescence		
Cell#	Mean	SD	SD/mean	Mean	SD	SD/mean	Mean	SD	SD/mean
1	309	21.5	0.0696	313.02	15.82	0.0505	311.33	12.55	0.0403
2	370	15.11	0.0409	395.52	15.02	0.038	405.17	16.82	0.0415
3	298	11.61	0.039	336.67	17.59	0.0522	337.33	14.21	0.0421
4	376.5	17.65	0.0469	396.76	14.73	0.0371	406.13	14.76	0.0363
5	328.5	15.99	0.0487	405.93	16.23	0.04	427.5	30.76	0.0719
6	413.3	25.67	0.0621	445	26.57	0.0597	471	28.07	0.0596
7	438	14.32	0.0327	420	12.57	0.0299	451.02	10.85	0.0241
8	307.5	21.21	0.069	353.48	25.16	0.0712	358.95	26.22	0.0731
9	404.4	26.02	0.0643	419.74	25.49	0.0607	442.35	31.92	0.0722
10	383.7	21.61	0.0563	434.17	36.79	0.0847	437.69	23.2	0.053
**Mean = **	362.9*	**19.07**	**0.0529**	392.03*^#^	**20.6**	**0.0524**	**404.85^#^**	**20.94**	**0.0514**
**SD = **	49.38	4.877	0.0132	43.656	7.628	0.0171	52.618	7.983	0.0172
**SEM = **	15.62	1.542	0.0042	13.805	2.412	0.0054	16.639	2.525	0.0055

Mean values of CL slightly, but statistically significantly, increased, * and ^#^ P<0.005 (pared T test). Experimentally measured sequence of consecutive CL values (‘tachogram’) for cell #2 is shown in Figure 1.

At least 20 spontaneous cycles were recorded for each CL measurement. Statistical comparison of the results of three experimental measurements was performed using a paired T-test. After Fluo-4AM loading into cells, the CL of cell spontaneous beating slightly increased from 362.9±15.6 ms (mean±SEM) to 392.0±13.8 ms (P<0.005). During measurements of Ca^2+^ transients (when Fluo-4 was excited by blue light), the CL further slightly increased to 404.9±16.6 ms (P<0.005). However, the coefficient of variation of CL (defined as 100%×(CL standard deviation)/(CL mean)) before loading in red light, after loading in red light, and after loading in blue light did not significantly differ and was 5.29%, 5.24%, and 5.14%, respectively (Table 1). Thus, under our experimental conditions (1) in response to Fluo-4 loading and blue light excitation the rate of spontaneous beating slightly decreased but the intrinsic CL variability remained almost unchanged; (2) any effect of possible photo-damage was shown to be negligible.

In longer recordings the sensitivity of cells to phototoxicity notably varied on a cell-to-cell basis. The effect of phototoxicity (i.e. increase in CL and CL variability) becomes notable on average after about 20 seconds of excitation of Fluo-4 (not shown). However, some cells were able to keep their 10 second moving average of CL and CL variability up to 40 seconds. On the other hand, in the worst case scenario, phototoxicity was clearly observed after about 12 seconds of excitation. We did not further examine the basis for different cell sensitivity to phototoxicity, but rather used all available data limited by 10 seconds of recording, which was sufficient to address the specific aims of our study.

### Electrophysiology and Synchronization of Simultaneous Recordings

A perforated patch-clamp technique was used in selected experiments to simultaneously measure APs and 2D Ca^2+^ signals. Membrane patch perforation was achieved using β-escin added to the patch pipette solution, as described elsewhere [11]. The patch pipette solution contained in mmol/L: 120 K-gluconate, 5 NaCl, 5 Mg-ATP, 5 HEPES, 20 KCl, 3 Na_2_ATP (pH adjusted to 7.2 with NaOH). Axopatch 200B and pCLAMP software (Molecular Devices, USA) were used for data acquisition. The liquid junction potential was corrected by tuning “the pipette offset” each time before touching the cell surface by setting the patch pipette current to zero, with the “command” voltage being set to “zero”. AP measurements were performed in a standard zero-current-clamp (fast mode) using a gap-free acquisition protocol, with a sampling interval of 0.1 ms. Ca^2+^ and AP recordings were synchronized by application of a short (20 ms) voltage pulse that was simultaneously recorded by the high-speed Hamamatsu camera (via an LED flash) and by pCLAMP (in a separate acquisition channel on the Digidata).

### Bathing Solution and Temperature Control

All Ca^2+^ and AP measurements were performed at 35°±0.1°C (500 µl chamber volume). Temperature was controlled by an Analog TC2BIP 2/3Ch bipolar temperature controller from CellMicroControls (USA). This heated both the glass bottom of the perfusion chamber and the solution entering the chamber (via a pre-heater). The physiological (bathing) solution contained (in mM): NaCl 140; KCl 5.4; MgCl_2_ 2; HEPES 5; CaCl_2_ 1.8; pH 7.3 (adjusted with NaOH).

### Data Analysis

Both APs and Ca^2+^ waveforms were analyzed by a custom-made (Victor Maltsev) program built with Delphi-7 developing software. DD rate was calculated as the difference between Maximum Diastolic Potential (MDP) and an AP threshold (set to −40 mV), divided by the time span required for the membrane to depolarize from the MDP to −40 mV. Ca^2+^ signal waveforms (with acquisition time on the abscissa, and fluorescence intensity on the ordinate) were generated from movies (i.e. stacks of consecutive images) of Ca^2+^-dependent fluorescence of Fluo-4 by calculating the spatial average fluorescence of each image within a user defined ROI (Region Of Interest). ROIs were chosen to cover either the entire cell perimeter (in order to assess total integrated cell Ca^2+^ signal) or a specific region within SANC (to measure characteristics of individual LCRs within specific loci).

In a comprehensive study involving hundreds of isolated SA node cells, Lyashkov et al. [12] failed to demonstrate significant differences in small (presumably central) and large (presumably peripheral) SANC in terms of labelling of RyR and SERCA2, labelling of NCX1, or in average AP characteristics, LCRs, or spontaneous cycle length. Therefore, we have not differentiated between SANC isolated by identical techniques on the basis of their LCR characteristics and in our statistical analysis we plot an average LCR period (i.e. a characteristic of the entire LCR ensemble) calculated for each cycle in all measured (stereotyped) cells and statistically tested whether it is linked to the length of that cycle (CL).

### Detection of LCRs in 2D Image Sequences

**Figure 2:**
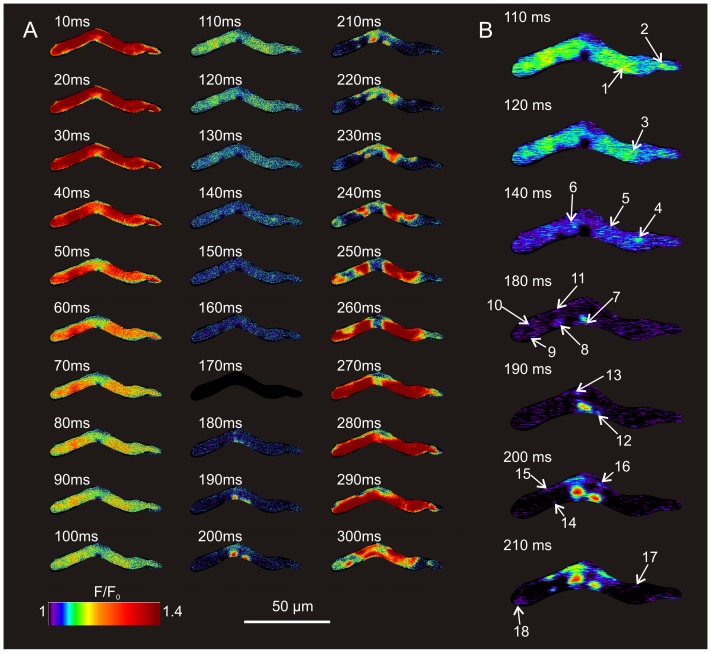
Spatiotemporal dynamics of Ca^2+^ signals in 2D. A: Frame-by-frame sequence of a single diastolic interval. The peak of the AP-induced whole cell Ca^2+^ transient is demonstrated in the first frame (t = 10 ms) and the latter frames (∼t = 280 ms), with maximal Fluo-4 fluorescence (dark red) present homogenously throughout the cell in these frames. The subsequent development of LCRs can clearly be seen in these enhanced images, beginning at time 110 ms. The nadir of global Ca^2+^ fluorescence occurs at 170 ms, and is represented by a complete lack of signal due to normalization of the fluorescent signal at this point. B: A more detailed view of the frames in panel A during which critical LCRs emerge, with each of the individual LCRs (1 through 18) labeled with a white arrow at its time of onset. Note: Twenty spontaneous Ca^2+^ cycles of this cell are also presented in Movie S1.

**Figure 3:**
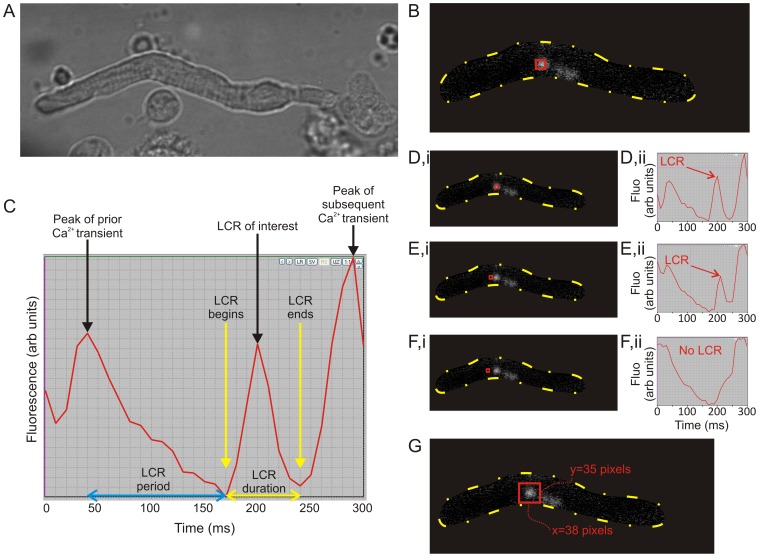
Detection of the fine spatiotemporal structure of a local Ca^2+^ release (a representative example). A: An SA node cell image in transmitted light. B: An enhanced still image of same cell demonstrating several discrete LCRs. The outline of the cell is emphasized with the broken yellow line. The red ‘hunting’ square is located over the LCR with the most marked fluorescent signal. C: The time course of the Ca^2+^ signal in the ‘hunting square’ (red square in panel B). The plot demonstrates 2 separate consecutive Ca^2+^ transients ‘book-ending’ the discreet peak in Ca^2+^ fluorescence caused by the LCR itself. LCR duration (shown by the yellow double-headed arrow) can be ascertained by looking at the beginning and end times of the discrete fluorescence peak associated with the LCR. The LCR period was measured as the difference in time between the peak of the prior AP-induced Ca^2+^ transient and the onset of the LCR (blue double-headed arrow). D–F: illustrate how the LCR size is calculated using the semi-automated technique. The ‘hunting square’ region of interest (red square) is made small relative to the LCR. When the square is positioned directly over the LCR (D,i), the peak in fluorescence caused by the LCR is obviously apparent in the live, automatically updating graph (D,ii). As the hunting square is gradually advanced away from the center of the LCR (in a leftward direction in this case, E,i) the amplitude of the spike in Ca^2+^ fluorescence decreases (E,ii). With continued advancement of the square away from the LCR (F,i), the peak associated with LCR disappears completely when the region-of-interest hunting square is no longer over any part of the LCR associated fluorescence (F,ii). The exact location where fluorescence associated with the LCR disappeared is marked, and the process is repeated in a total of eight directions around each LCR, until it is possible to draw a perimeter identifying the border of the LCR, and calculate its approximate dimensions in 2D (panel G).

We used a semi-automated method to detect LCRs in a given spontaneous cycle using a custom-made image analysis program (developed by Victor Maltsev). Figure 2 illustrates a single diastolic period in which the program processed the fluorescence related to diastolic Ca^2+^ releases to maximize their visibility. Figure 3 illustrates the process used to define the characteristics of individual LCRs in a typical SANC, shown under light microscopy in Figure 3A. First, the ROI was selected to include the entire cell perimeter (yellow broken line, Figure 3B), and the total, spatially-averaged cell Ca^2+^ signal was calculated. The cycle of interest was defined as the period of time from one peak Ca^2+^ transient to another. The frame with minimum fluorescence within that cycle was considered as the Ca^2+^ signal nadir (F_0_) and used further for normalization of all the other images in the stack. After normalization and contrasting maximum intensity (max F/F_0_) with a color scheme, the LCRs (events) could be observed as discrete, temporary and local increases in fluorescence intensity within the cell perimeter (Figure 2). To separate an LCR from the noise, and to determine its spatiotemporal borders, we defined the LCR as a group of adjacent (neighboring) pixels whose amplitudes were greater than one standard deviation above the mean value of the background signal (Figure 3). We measured individual characteristics of LCRs, including their duration, maximum attained amplitude, size, and LCR period (i.e. timing of onset relative to the prior AP-induced Ca^2+^ transient peak) - see Figure 3C. The process of defining the size of an LCR is outlined in detail in Figures 3D–G. Some of the later occurring LCRs fused with the subsequent AP-induced Ca^2+^ transient; where this was the case, the LCR maximum amplitude was taken to be that reached at the end of the diastolic period (end of image stack).

## Results

### A General Pattern of Ca^2+^ Dynamics in Spontaneously Beating SANC is Revealed by High-speed 2D Camera Recordings

Ca^2+^ signals exhibit two discrete phases within each spontaneous cycle (see example in Movie S1 and Figure 2). The first (diastolic) phase consists of multiple local Ca^2+^ signals (‘LCRs’), which appear relatively synchronously in geographically diverse locations within the cell, and begin to propagate locally. Some LCRs terminate and disappear, while others continue to grow in size, merging into the second (systolic) phase of Ca^2+^ release, observed as a strong, high-amplitude global cytosolic Ca^2+^ transient (CaT) induced by the occurrence of the subsequent AP. Thus, the diastolic LCR ensemble develops progressively until interrupted by the subsequent AP-induced CaT’s upstroke. In turn, as the CaT decays, a new cycle of diastolic LCR ensemble emerges once more, recapitulating the events of the preceding cycle.

### Beat-to-beat Variations of DD Rate are Linked to Variations in Timing of LCR Occurrence

**Figure 4:**
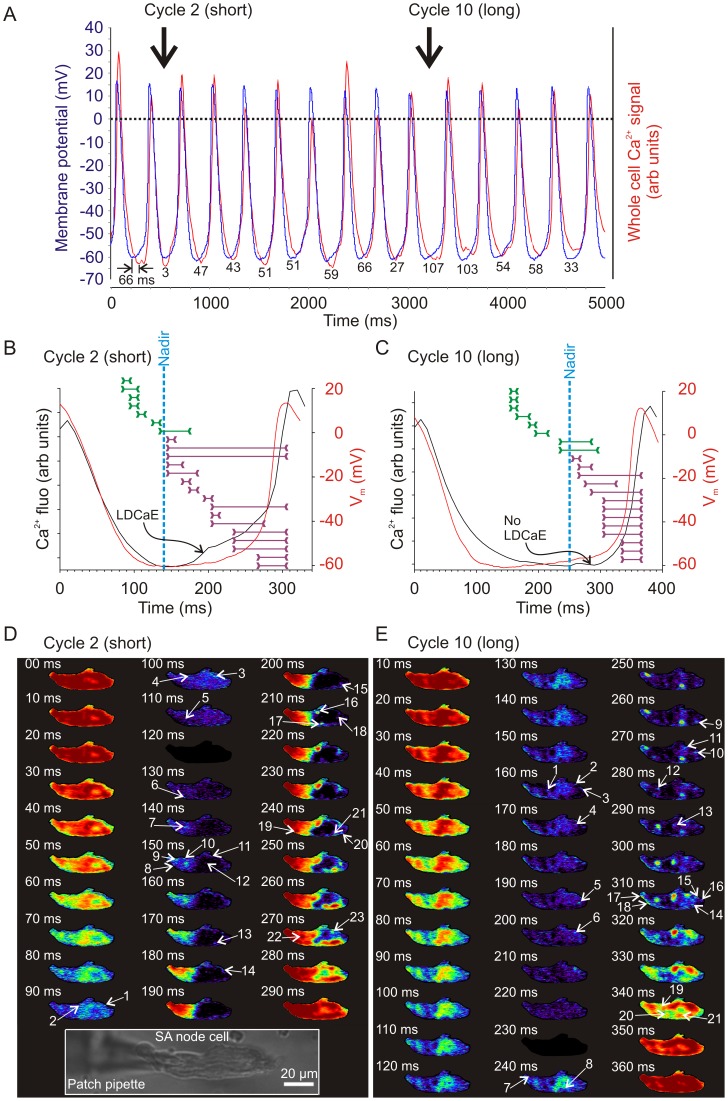
Different dynamics of local Ca^2+^ signals and membrane potential in short and long spontaneous cycles. A: An example of simultaneous recordings of the whole cell Ca^2+^ fluorescent signal by the fast 2D camera (integrated spatially over the cell) and the membrane potential recorded by perforated patch clamp. In this example, 14 consecutive cycles from a single representative rabbit SANC are shown. The numbers below each cycle reflect the time interval (in ms) between the MDP and the global Ca^2+^ signal nadir. In general, short cycles were associated with small differences between MDP and nadir, whereas long cycles exhibited large differences between MDP and nadir. B,C: A more detailed analysis of a typical short (#2, panel B) and long (#10, panel C) cycle dissected out from the continuous recording shown in panel A. The diastolic intervals are shown, along with individual LCR occurrence timing, illustrated as horizontal ‘span’ lines demonstrating the duration of each individual LCR from onset to disappearance. The global Ca^2+^ nadir (dashed blue lines) and LDCaE signals (black arrows) formed by the LCRs markedly differ in the two examples. In the short cycle, the global Ca^2+^ nadir occurs early, and the LDCaE is prominent, whilst the opposite is true for the long cycle. D,E: Frame-by-frame sequences of enhanced fluorescent images (frames) to illustrate spatiotemporal dynamics of individual LCRs (white arrows, with numbers indicating the number of the LCR within that diastolic interval) and the AP-induced CaT in the short and long cycles illustrated in panels B,C respectively. Inset shows a light microscopic image of the cell with the attached patch pipette. Note: Fourteen spontaneous cycles of simultaneously recorded APs and Ca^2+^ transients in this cell are also presented in Movie S2.

We explored how LCR activity during diastole is linked to pacemaker APs via simultaneous perforated patch-clamp and 2D camera recording. An example of a typical simultaneous recording from a selected cell is shown in Movie S2 and Figure 4. Short cycles (e.g. cycle #2 in Figure 4) exhibited a steep diastolic depolarization (red curve), coincident with a later, pronounced ‘bump’ in the whole cell diastolic Ca^2+^ signal (black curve).

The diastolic Ca^2+^ release signal (‘bump’ in Figure 4B) appears to be similar to that observed in previous confocal line-scan studies (following integration of the local Ca^2+^ signal for several cycles [13]), and has also been noted in each cycle in whole SA node Ca^2+^ recordings, where it has been termed the ‘Late Diastolic Ca^2+^ Elevation’ [14] (‘LDCaE’, indicated by the black arrow in Figure 4B). Longer cycles, contrastingly, (e.g. cycle #10 in the given example) demonstrate a much flatter diastolic phase to their membrane potential, and a less pronounced LDCaE in the whole cell Ca^2+^ fluorescence recording (Figure 4C, black arrow).

To illustrate the timing of the onset of DD increase with respect to diastolic Ca^2+^ release, we simultaneously plotted two derivatives (Figure S1): for membrane potential (dV/dt) and for the global Ca^2+^ signal (dF/dt). In a representative short cycle dV/dt increase closely follows dF/dt increase (inset), reflecting diastolic Ca^2+^ release flux and its effect on membrane potential (likely via NCX current [8], [9]). In contrast, a longer cycle showed a “flat” time course for both derivatives during almost the entire DD span, i.e. with no evidence of Ca^2+^ release, and with dV/dt remaining relatively small (close to the zero line).

Comparison of the finer temporal structure of the total diastolic Ca^2+^ signal (i.e. individual LCRs) of the short and long cycles in 2D images (Figure 4D,E respectively) was undertaken to elucidate the relationship of the LCR ensemble to CL. We identified the initiation of each LCR within cycles (shown by arrows in respective panels in Figure 4B,C), and then constructed representative lines of “LCR span”, delineating the time course of each LCR from beginning to end. When these LCR span lines are superimposed onto traces of the whole cell Ca^2+^ transient and simultaneously recorded membrane potential (Figure 4B,C), it becomes apparent that LDCaE are formed by an ensemble LCR signal that appears to be more synchronized within shorter cycles than in longer cycles. Indeed, in shorter cycles, more LCRs overlap with each other during DD (including more overlapping in earlier diastolic phases close to the MDP). Longer cycles, in contrast, display less synchronization of LCRs. Indeed they display several diastolic frames with only single LCR occurrences, and some frames in which no LCRs occur at all (e.g. frame 180 ms in Figure 4E, cycle 10). A comparison of the number of overlapping LCR events relative to time from prior peak of Ca^2+^ transient is shown in Figure S2. It can be seen from this figure that the LCRs of the shorter cycle occur earlier, with a substantially higher number of early occurring overlapping LCRs compared to the longer cycle, in which LCRs only begin to overlap at a time significantly later in the LCR period. This suggests that early coordinated production of LCRs is an important determinant of a short cycle length.

It is important to note that the observed cycle-to-cycle variation in LCR synchronization results not only in variation in the prominence of LDCaE, but also in variation of the timing of LDCaE onset. In the example shown in Figure 4B,C, the LDCaE begins to emerge 170 ms after the CaT peak in the short cycle, but only at about 300 ms in the long cycle. The LDCaE actually represents the rising integral signal of the diastolic LCR ensemble, and therefore it begins when the ensemble of waxing LCR activity converts the global Ca^2+^ trend from decay into rise, so forming a nadir (i.e. a minimum), shortly thereafter heralding LDCaE occurrence.

**Figure 5:**
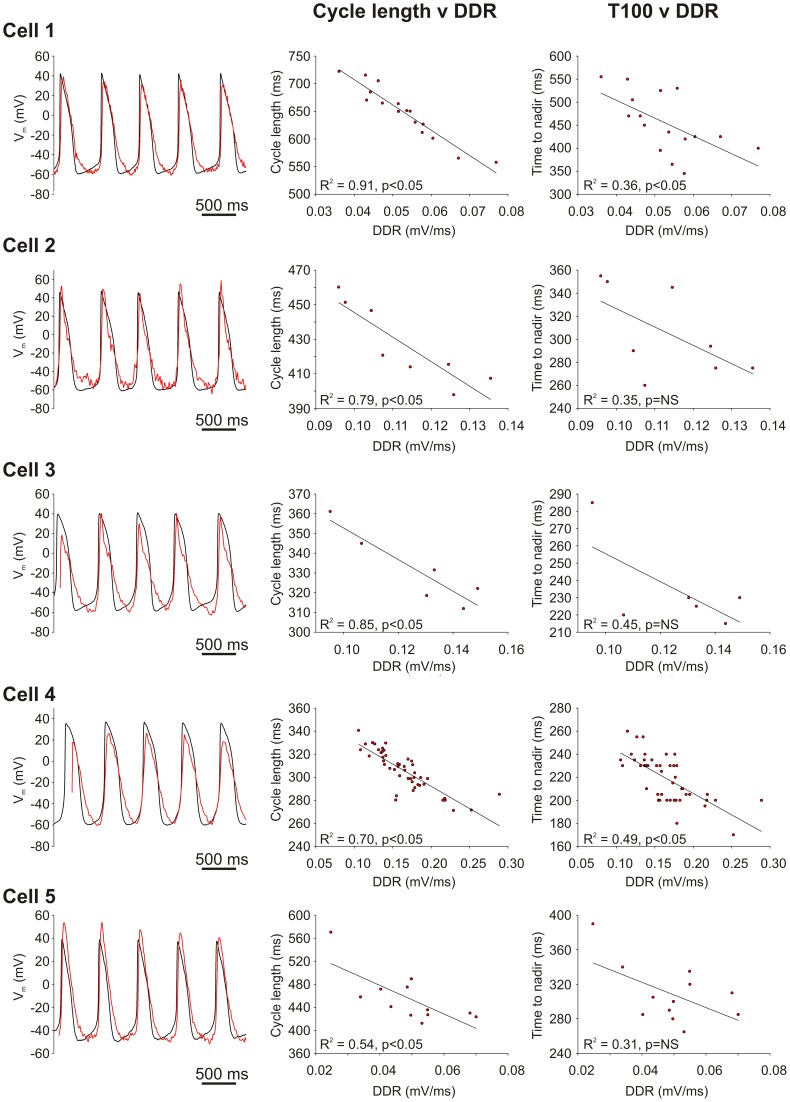
The effect of DD rate on CL, and the effect of time to nadir of Ca^2+^ transient on DD rate. Original waveform data from simultaneous recordings of Ca^2+^ signals (red) and membrane potential (black) in five representative SANC are shown in the left-hand panels. CL is closely predicted by DD rate (middle panels). DD rate, in turn, is closely predicted by time from prior peak to the nadir of the whole cell Ca^2+^ signal (referred to as ‘T_100_’ in the figure, right-hand panels). The R^2^ value and p-value of the linear regression lines are shown on the respective graphs, with statistically significant relationships being defined as those where p<0.05 for the linear regression line. p = NS refers to a non-statistically significant p-value, i.e. one greater than 0.05.

Since the timing of the global Ca^2+^–dependent fluorescence nadir represents the impending onset of LDCaE that is associated with DD acceleration, we further explored the importance of this parameter with regards to beat-to-beat variations in DD rate and CL. Figure 5 demonstrates results from five representative cells with simultaneously measured V_m_ and Ca^2+^ fluorescence (left-hand column shows the raw data from these cells). It can easily be seen that the DD rate reproducibly predicts CL in all of these cells (central column, with statistical significance (p<0.05 of the linear regression line) in each of the individual cells); similarly DD rate is predicted by the ‘time-to-nadir’ (i.e. the time period between the occurrence of the prior peak Ca^2+^ transient and the following minimum (nadir) of the spatially averaged whole cell Ca^2+^ signal; statistical significance (p<0.05) for this relationship is present in only two of the five represented cells, though the numbers of cycles studied in the cells in which statistical significance is absent is relatively few).

### Characteristics of LCRs: Early vs. Late in 2D: Number, Size, Duration

We further explored the characteristics of the entire LCR ensemble and determined:

1. the total number of LCRs per cycle
2. the ‘LCR period’, i.e. the specific time of each LCR occurrence with respect to the prior global Ca^2+^ signal peak
3. ‘LCR duration’, i.e. how long each LCR lasts for.

**Figure 6:**
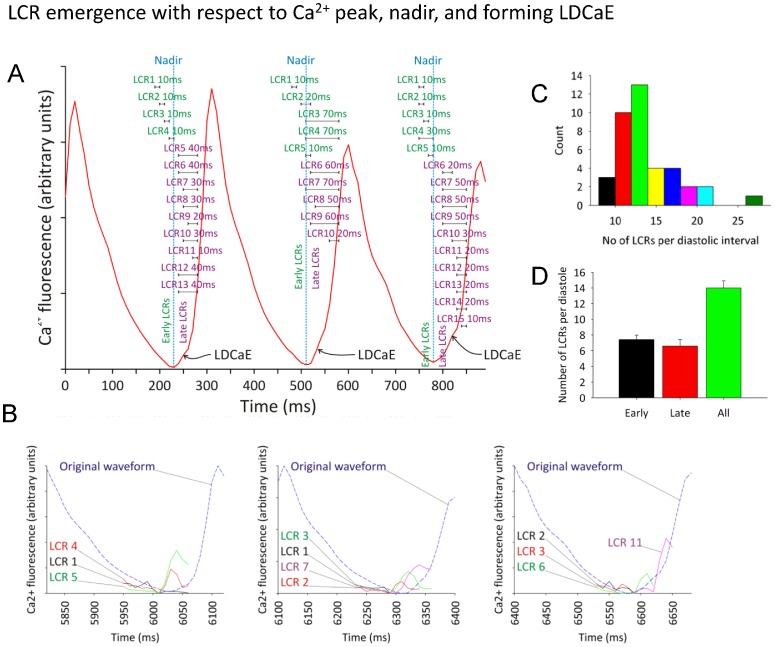
LCR emergence with respect to the global Ca^2+^ transient peak, global Ca^2+^ nadir, and formation of ‘Late Diastolic Ca^2+^ Elevation’ (LDCaE). A: Examples of how the whole cell global Ca^2+^ fluorescence signal recorded (red waveform trace) varies with time over three arbitrarily selected consecutive diastolic periods from a single cell. It can be seen that there are approximately rhythmical peaks and troughs in the spatially-averaged whole cell Ca^2+^ fluorescence. Horizontal ‘span’ bars represent the duration of individual LCRs, from their appearance to disappearance. LCRs are further categorized into early (labeled in green) and late (labeled in magenta), depending on when they begin with respect to the whole cell spatially averaged Ca^2+^ fluorescence trace (an early LCR begins *before* global Ca^2+^ nadir (blue broken line), a late LCR begins *after* the nadir). The waxing LCR number and intensity with time leads to the LDCaE (Late Diastolic Ca^2+^ Elevation), and a prominent increase in rate of rise of Ca^2+^ fluorescence (black curved arrows). B: Illustration of individual LCR signals. In each of the three diastolic periods illustrated in A, the global Ca^2+^ transient signal (blue dashed line) is plotted along with the local Ca^2+^ fluorescence signal for selected early-, average- and late-occurring local LCRs, to illustrate how the local Ca^2+^ signal varies with respect to the whole cell signal. C: Bar chart to demonstrate how LCR number varies in all of the diastolic intervals studied (data from 9 cells). D: Bar chart demonstrating the mean number of early and late LCRs per diastole, showing that numbers of early and late diastoles are approximately equal across all the diastolic intervals studied.

On average (n = 9 cells), under basal conditions, SANC generated 13.2±3.7 LCRs per cycle. Note that the distribution of LCR number per diastole is not Gaussian, but rather is skewed towards larger values (Figure 6C). In contrast to the present dogma that LCRs represent purely a late DD mechanism (i.e. occur close to the next CaT), our 2D recordings revealed that some LCRs, in fact, begin to occur relatively early within the diastole, some on the *descending* part of the prior CaT, at about 50% of each spontaneous cycle duration (if measured from peak-to-peak of consecutive CaTs), i.e. relatively long before the subsequent CaT. Figure 6A quantitatively represents this finding by portraying occurrence times of the entire LCR ensemble with respect to the global Ca^2+^ signal (i.e. the spatial Ca^2+^ average) for 3 consecutive diastolic periods. The plot shows LCRs once more as ‘span lines’, delineating their duration from beginning to end, superimposed on the whole cell Ca^2+^ transient. Figure 6B shows the overlapped time series of several representative LCRs for each of the 3 diastoles in Figure 6A with respect to whole cell Ca^2+^ transient (labeled as “original waveform”).

About half of all LCRs (53%, Figure 6D) occur at or before the global CaT nadir (marked by blue vertical line). These events are termed “early LCRs”, while those occurring after the nadir of the CaT are termed “late LCRs”. Note that some early LCRs terminate at or prior to the nadir of the global Ca^2+^ signal. Contrastingly, many late LCRs do not terminate as such, but rather continue to propagate until they are engulfed by the overwhelming effect of the on-rushing AP-induced CaT.

**Figure 7:**
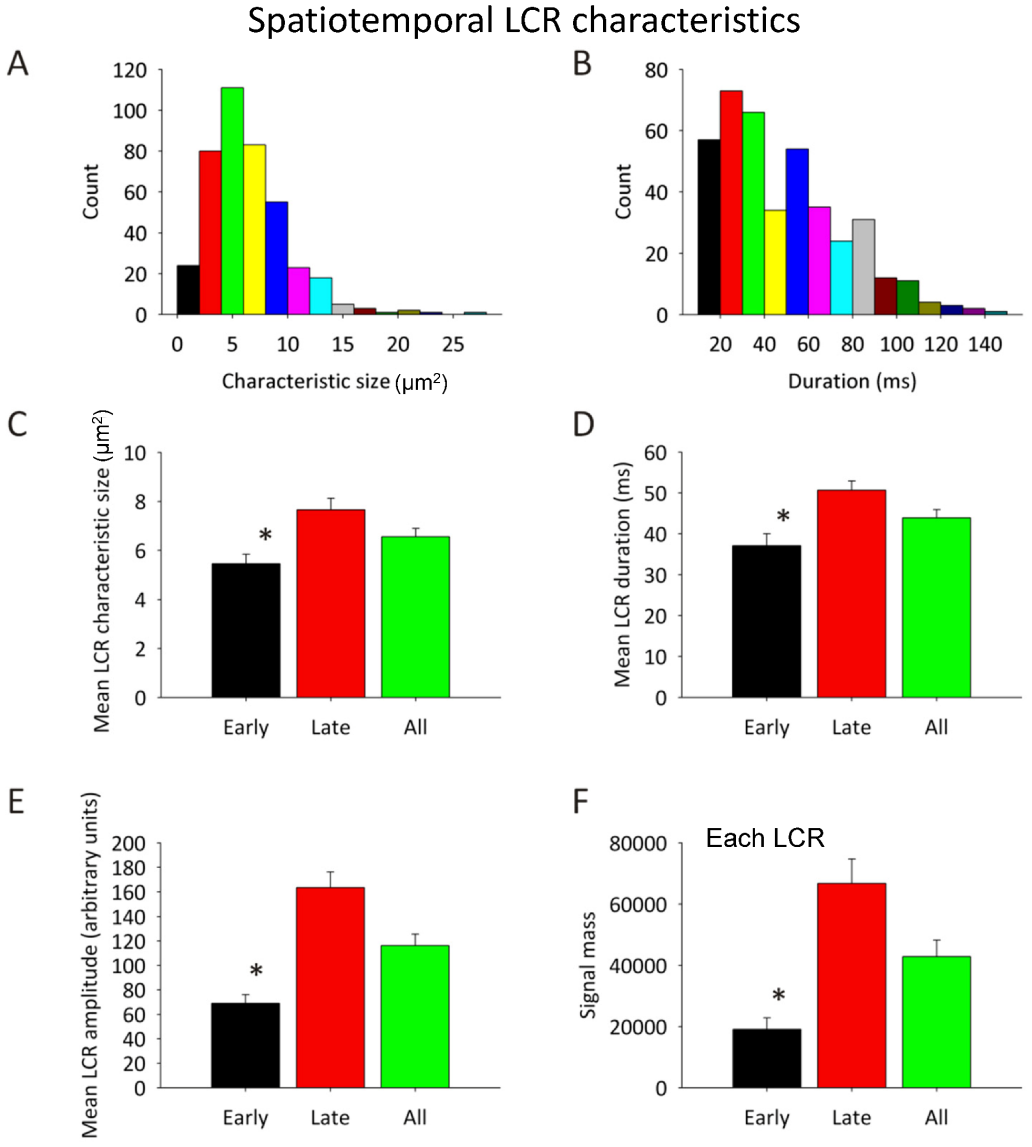
Spatiotemporal LCR characteristics in 2D. A,B: Histograms (data from 9 cells) demonstrating the spread in size (in µm) and duration (in ms) of LCRs. C–F: Bar charts comparing mean LCR characteristic size, duration, amplitude and calculated signal mass, with distinction drawn between early (black bars) and late (red bars) LCRs, with the mean of all early and late LCRs together included on the figures (green bars). * = p<0.05 on 1-way ANOVA between early and late LCRs.

Consistent with the complex pattern of spatiotemporal dynamics of LCRs described above, our overall analysis of 2D recordings in nine SANC revealed that LCRs do indeed vary in size (assessed as LCR area, mean 7.1±4.2 µm^2^) and duration (44.2±27.1 ms) (see respective histograms in Figure 7A,B). One reason for such intrinsic variation in LCR size and duration is that the characteristics of the aforementioned “early” LCRs differ markedly from those of “late” LCRs. Early LCRs are significantly smaller in size (Figure 7C), shorter in duration (Figure 7D), less intense in amplitude (Figure 7E) and have an overall smaller Ca^2+^ signal mass (Figure 7F) than their later occurring counterparts.

### Timing of LCR Occurrence Predicts Variations of Cycle Length

**Figure 8:**
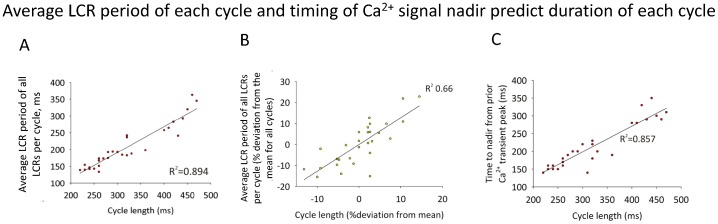
The timing of LCRs is strongly related to cycle length (data from 9 cells). A: The relationship between mean LCR period and cycle length, demonstrates that when the mean period of LCRs is long, then the cycle’s length will be long, and when the mean LCR period is short, then the cycle’s length will be short. B: Data in A expressed as a % deviation from mean of the individual cell’s data. It is important to note that we did not separate or group individual cells by their LCR (or any other) characteristics (see more about our “stereotyped” cell assumption in Methods, section “Data Analysis”). C: The relationship between time from peak of the global Ca^2+^ transient to nadir (T_100_) and cycle length, demonstrating that there is also a strong relationship between the rate of waning of the Ca^2+^ transient, eventually forming a nadir, and that cycle’s length. The more rapidly that the waning whole cell Ca^2+^ transient ‘turns’ to begin to wax (effected by the growing LCR ensemble signal mass), the shorter the cycle length is likely to be. The data in panels A, B, and C are described by the linear regression model each with statistical significance of p<0.001 and with R^2^ shown on each plot.

We found that the mean time of LCR occurrence (i.e. mean LCR period of the LCR ensemble) within a given cycle predicts the length of that cycle (Figure 8A,B, for both absolute values and deviations from the mean over all cycles, respectively). This suggests that the LCR ensemble timing plays an important role in determining CL, and hence, cycle-to-cycle length variations that are witnessed in recordings from individual isolated SANCs.

The importance of waxing LCR activity in determining CL is also evidenced by the close and proportional relationship between CL and the timing of the global Ca^2+^ nadir (Figure 8C). The nadir can be conceptualized as the time at which spontaneous growth or ‘waxing’ of the integral of Ca^2+^ release (generated in diastole by LCRs) overcomes the general prior trend of decreasing, or ‘waning’, global cell Ca^2+^ signal due to fading of the prior AP-induced Ca^2+^ transient. In other words, the finding that the time at which the LCR ensemble Ca^2+^ release causes a nadir and initiates the LDCaE is related to cycle length represents further evidence that the timing of LCRs contributes to CL determination.

## Discussion

### Importance of Ca^2+^ for SANC Automaticity on a Beat-to-beat Basis

We have developed a new fast 2D imaging technique in SANC that allows for monitoring of local Ca^2+^ signals arising from the *entire* cell (rather than only signals within a restricted linescan region, as is the case in more traditional confocal imaging techniques). Our results provide direct, novel evidence of the cycle-to-cycle variations in local cell Ca^2+^ regulation, and the importance of these Ca^2+^ variations in cycle-to-cycle length variation in isolated SANC. Specifically, using a fast 2D camera to capture the total cell Ca^2+^ dynamics (including both AP-induced Ca^2+^ transients alongside the diastolic LCRs), we have, for the first time, directly linked the characteristics of the entire ensemble of LCRs to DD rate and CL on a beat-to-beat basis.

### Evolution of LCRs in 2D

The number of LCRs per cycle detected by 2D imaging of Ca^2+^ signals was substantially less than estimated from line scans in previous studies (∼14 (Figure 6C) vs. ∼40 [9]). One possible reason for overestimation in prior studies is that the position of the scan line was usually set by the experimenter to cross the cell area with abundant LCR activity (indeed, it would make no sense to study LCRs in line scans without LCRs present) and this activity was then extrapolated to estimate the total LCR number (per cell, per cycle). In contrast, our new 2D imaging technique is capable of accurately and simultaneously recording Ca^2+^ signals from the entire cell area, including those areas with and without LCR activity.

The surprisingly fewer LCRs per cycle, combined with the previously established major contribution of LCRs to the DD, places a critical importance upon intrinsic variation in characteristics of each of the few LCRs for their combined impact on the DD, and ultimately upon the spontaneous CL. This idea was confirmed by our finding that CL variation was closely predicted by the preceding variation in the respective average LCR period in each given cycle (Figure 8A,B). Our results also refute the previously held dogma that LCRs are mostly ‘late’ diastolic events. This dogma, in fact, has a technical basis, because whole cell and SAN tissue recording captures a *total* Ca^2+^ signal (lacking the ability to resolve the fine structure of individual LCRs), so that the observed global late diastolic Ca^2+^ elevation and its importance have been logically associated with “late” LCR activity. Our study demonstrates, however, that many LCRs occur close to the MDP (Figure 4B,C) and well before the global Ca^2+^ nadir (heralding the LDCaE onset, Figure 6A). In fact, the absolute number of early and late LCRs are almost the same (Figure 6D). We have also shown, for the first time, that the characteristics of these early LCRs substantially differ from those occurring later during diastole: early LCRs are smaller in size and shorter in duration and have a smaller signal mass (Figure 7F). Thus, the evolution of LCRs during DD represents a dynamic process, starting from small and short-lived individual sparks, and culminating in large-sized LCRs that merge into the CaT, as conditions for spontaneous Ca^2+^ release activation wax (e.g. SR Ca^2+^ refilling, L-type Ca^2+^ channel activation, and local Ca^2+^ induced Ca^2+^ release). While logically it is tempting to assign the role of early LCRs to that of priming the subspace for further stronger LCR activity, their exact role, mechanisms and importance of the discovered dynamics of LCR characteristics need to be further examined. With respect to late LCRs, these larger and longer LCRs would be expected to form a stronger integrated signal (i.e. signal mass, Figure 7F), underlying the observed LDCaE seen in the whole cell Ca^2+^ signal (Figure 6A).

### Mechanisms of LCR-linked CL Variations

Because the average LCR period among many LCRs and many AP cycles at a given steady-state of AP firing determines the average timing of LCR activation of NCX inward current during DD, the LCR period is tightly coupled to the average steady-state CL, with the coupling being preserved in numerous different perturbations of SANC function [4]. In a given steady state, however, the LCR occurrences introduce substantial noise into the amplitude of DD in SANC [9]. Further, numerical modeling simulations have demonstrated that this noise could account for beat-to-beat variability of spontaneous AP CL observed experimentally (Figure 7E of [9]). However, the ability to identify and record all of the local Ca^2+^ signals within a single SANC (present study) is crucial with respect to understanding how their integration or ensemble behavior is coupled to the AP on a beat-to-beat basis. We observed that CL fluctuations are linked, in large part, to the mean time at which stochastic LCRs emerge in each cycle, ultimately forming the LDCaE, which in turn affects the DD rate (Figure S1). The effect of LCRs (and their LDCaE) upon DD rate, in turn, is produced via activation of the NCX current. Indeed, previous experimental and numerical modeling studies have demonstrated that each LCR generates a miniature NCX current, so that a roughly periodic, “noisy” LCR ensemble is capable of generating a roughly periodic, “noisy” ensemble of miniature I_NCX_, respectively [9]. These I_NCX_ fluctuations cause DD rate fluctuations, and ultimately CL fluctuations.

Our present finding, that stochastic fluctuation of Ca^2+^ releases is linked to CL fluctuations, does not, however, contradict the idea previously suggested by Wilders and Jongsma, that “beating irregularity” is linked to stochastic openings of sarcolemmal ion channels during DD [2]. The two mechanisms operate during DD, and may simply be complementary. It is crucial to note, however, that the idea of the ion channel-linked basis of CL fluctuations was formulated in 1993 and was based purely on computer simulations using an electrophysiological SANC model, reflecting the state of the pacemaker field at that time. The contributions of different ionic currents have since been substantially revised (see review [15]) and novel pacemaker mechanisms have been suggested, including those linked to LCRs (review [4]). In contrast to the 1993 computer simulation study of Wilders and Jongsma, the present study, for the first time, has measured the stochastic events (i.e. all LCRs or the LCR ensemble) within the entire cell, and thus the novel idea that individual CL fluctuations in isolated SANC are linked to fluctuations in Ca^2+^ cycling has herein been directly tested.

### Future Studies

Previous studies from our group have demonstrated a crucial role for SR Ca^2+^ refilling kinetics in the timing of LCR emergence [7]. Thus, in addition to the stochastic nature of RyR openings, another major determinant of LCR period fluctuations is likely to be beat-to-beat variations in SR Ca^2+^ refilling (i.e. SR Ca^2+^ pumping). On the other hand, SR Ca^2+^ refilling depends upon the amount of Ca^2+^ available for pumping (i.e. Ca^2+^ influx via I_CaL_), along with the state of phosphorylation of Ca^2+^ cycling proteins, which may also have local and time-dependent variations. These complex (“coupled-clock”) systems [4] ought to be further examined experimentally and by using numerical modeling (including local Ca^2+^ control [16]) to clarify the contributions of specific mechanisms underlying the beat-to-beat variations in LCR period found in the present study.

The magnitude of the impact of LCRs observed in the reductionist SANC model system upon cycle length in the multicellular, substantially more complex isolated sinoatrial nodal preparation, whole organ and *in vivo* mandates further study. One approach that has been recently developed is to directly measure Ca^2+^ signals within individual cells of intact heart tissue [17], including SA node [18]. Since normal heart rate variability exhibits complex fractal-like behavior, i.e. a self-similarity observed at different time scales [1], it will be interesting to clarify whether or not LCRs of cardiac pacemaker cells contribute to this complexity. Presently, at least two experimental studies point to a possible functional importance of LCRs in SA node tissue: 1) LCR activity detected in individual cells of SA node tissue increases in some types of cardiac arrhythmia [18]; 2) Late diastolic Ca^2+^ elevation, LDCaE (most likely produced by the LCR ensemble, see Figure 4) has also been detected in SA node tissue [14].

### Results Summary and Conclusions

This is the first study to quantify the fine spatiotemporal structure of Ca^2+^ dynamics in SANC, including the entire ensemble of individual LCRs, in 2D. We found that LCRs actually begin to appear very early within the cycle, i.e. overlapping with the decaying whole-cell CaT and occurring well before the late diastolic Ca^2+^ elevation observed in the global (whole-cell) Ca^2+^ signal previously measured in SAN cells and tissue. Intrinsic cycle length variability in single SANC is linked to beat-to-beat variations in the average period of individual LCRs each cycle. Thus, these new results together with our recent results using photo-flash-induced Ca^2+^ release [19] demonstrate Ca^2+^ regulation of cardiac pacemaker function on a beat-to-beat basis.

## Supporting Information

Figure S1 **Illustration of the timing of the onset of diastolic depolarization increase with respect to Ca^2+^ release in representative short (2) and long (10) cycles identified in panels B,C in main text** **Figure 4** **.** Panels show two derivatives: for membrane potential (dV/dt) and for the global Ca^2+^ signal (dF/dt). In the short cycle diastolic dV/dt increase follows dF/dt increase (inset), indicating that diastolic Ca^2+^ release accelerates diastolic depolarization. In contrast, a longer cycle showed “flat” time course for both derivatives during almost entire diastolic depolarization span, i.e. with no evidence of Ca^2+^ release and dV/dt remaining relatively small (close to zero line).
(JPG)

Figure S2 **A comparison of the number of overlapping LCR events relative to time from the peak of the prior AP-induced Ca^2+^ transient in the short and long cycles identified in panels B,C in main text** **Figure 4** **.** The number of overlapping LCR events in the shorter cycle (2) is illustrated by the red bars, while the number of overlapping LCR events in the longer cycle (10) is shown by the green bars.
(JPG)

Movie S1 **A representative example of original 2D recording of intracellular Ca^2+^ dynamics (Fluo-4 signals) for twenty pacemaker cycles in an isolated single rabbit SA node cell.** The movie consists of 575 frames (images) consequently recorded at a physiological temperature of 35°C by a high-speed Hamamatsu C9100-12 CCD camera with an approximate rate of 100 frames/second (5.75 s total duration). The movie is played 3 times slower vs. real time recording. Images have size 512×208 pixels (or 128×52 µm). The cell shown in this movie is also presented in Figures 2 and 3.
(MP4)

Movie S2 **A representative example of simultaneous recording of 2D intracellular Ca^2+^ dynamics (Fluo-4 signals, top panel) by a high-speed Hamamatsu C9100-12 CCD camera and action potentials (bottom panel) by perforated patch clamp in an isolated single rabbit SA node cell.** Middle panel shows simultaneous dynamics of the total Ca^2+^ signal (calculated total Fluo-4 fluorescence). The movie shows 14 pacemaker cycles (about 5 s total duration) recorded at a physiological temperature of 35°C. The movie is played about 4.6 times slower vs. real time recording. Images have size 512×160 pixels (or 128×40 µm). The cell shown in this movie is also presented in Figure 4.
(WMV)
