# Supplementary figures and images for: Beat-to-Beat Variation in Periodicity of Local Calcium Releases Contributes to Intrinsic Variations of Spontaneous Cycle Length in Isolated Single Sinoatrial Node Cells
Source: PLoS One. 2013 Jun 27;8(6):e67247. doi: 10.1371/journal.pone.0067247 (PMC3695077; doi:10.1371/journal.pone.0067247)

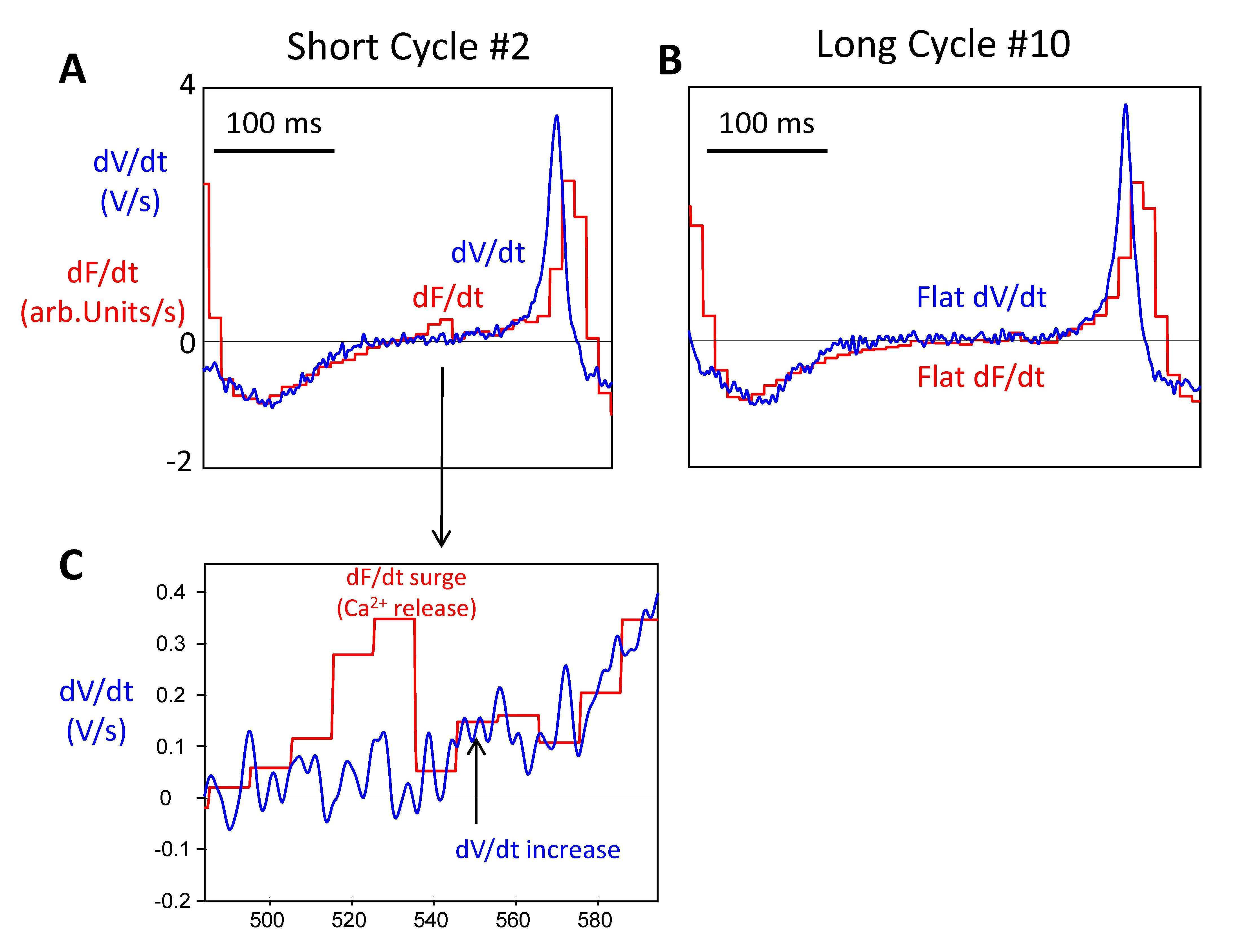

Supplement: Figure S1 — Illustration of the timing of the onset of diastolic depolarization increase with respect to Ca2+ release in representative short (2) and long (10) cycles identified in panels B,C in main text Figure 4 . Panels show two derivatives: for membrane potential (dV/dt) and for the global Ca2+ signal (dF/dt). In the short cycle diastolic dV/dt increase follows dF/dt increase (inset), indicating that diastolic Ca2+ release accelerates diastolic depolarization. In contrast, a longer cycle showed “flat” time course for both derivatives during almost entire diastolic depolarization span, i.e. with no evidence of Ca2+ release and dV/dt remaining relatively small (close to zero line). (JPG) [file pone.0067247.s001.jpg]

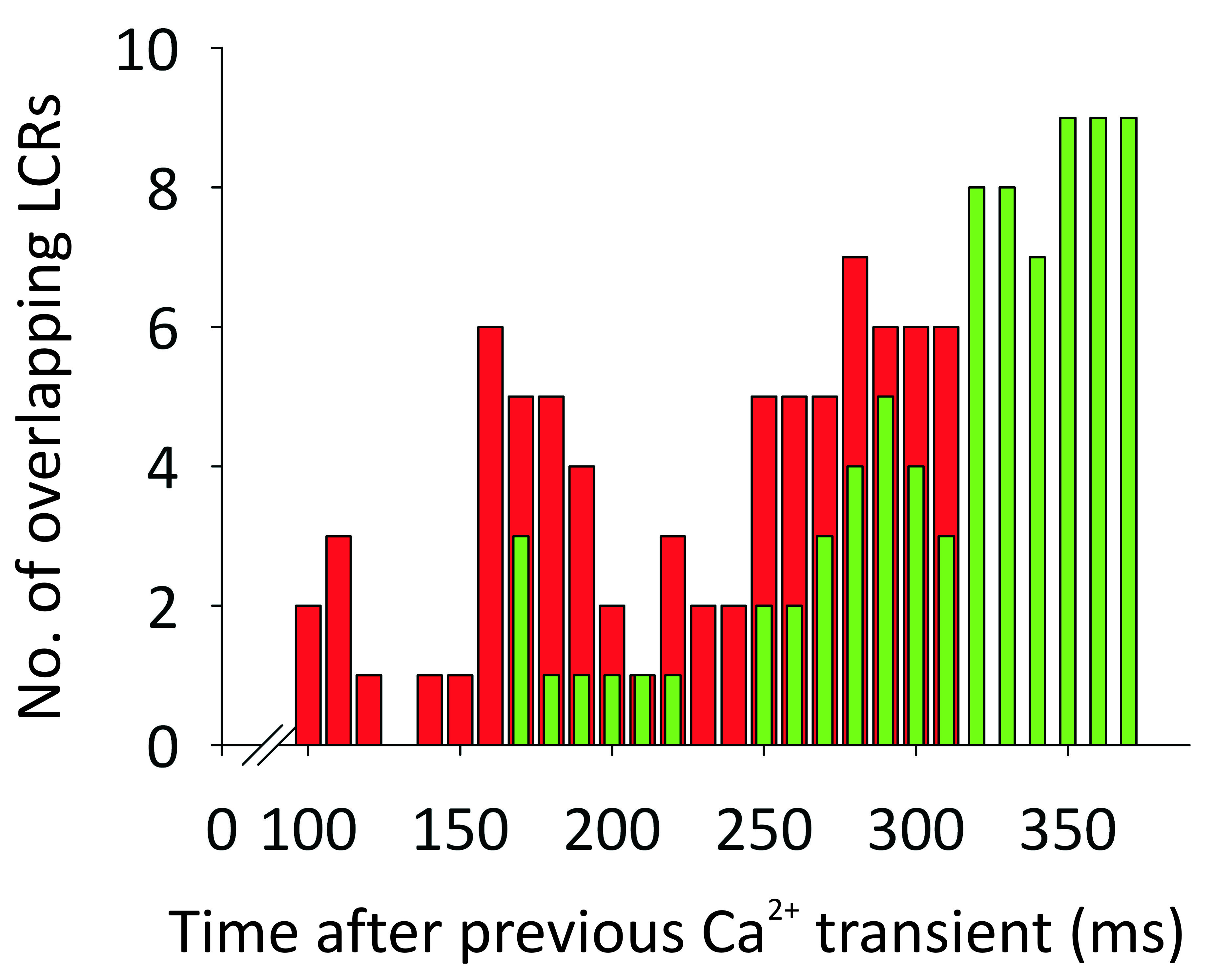

Supplement: Figure S2 — A comparison of the number of overlapping LCR events relative to time from the peak of the prior AP-induced Ca2+ transient in the short and long cycles identified in panels B,C in main text Figure 4 . The number of overlapping LCR events in the shorter cycle (2) is illustrated by the red bars, while the number of overlapping LCR events in the longer cycle (10) is shown by the green bars. (JPG) [file pone.0067247.s002.jpg]
